# Pomegranate extract decreases oxidative stress and alleviates mitochondrial impairment by activating AMPK-Nrf2 in hypothalamic paraventricular nucleus of spontaneously hypertensive rats

**DOI:** 10.1038/srep34246

**Published:** 2016-10-07

**Authors:** Wenyan Sun, Chunhong Yan, Bess Frost, Xin Wang, Chen Hou, Mengqi Zeng, Hongli Gao, Yuming Kang, Jiankang Liu

**Affiliations:** 1 Department of Nutrition and Food Security, School of Public Health, Xi’an Jiaotong University, Xi’an 710061, China; 2 Department of Physiology and Pathophysiology, School of Basic Medical Sciences, Xi’an Jiaotong University Cardiovascular Research Center, Xi’an Jiaotong University, Xi’an 710061, China; 3 Center for Mitochondrial Biology and Medicine, The Key Laboratory of Biomedical Information Engineering of Ministry of Education, School of Life Science and Technology and Frontier Institute of Science and Technology, Xi’an Jiaotong University, Xi’an 710049, China; 4 The Sam and Ann Barshop Institute for Longevity and Aging Studies, Department of Cellular and Structural Biology, University of Texas Health Science Center San Antonio, San Antonio, Texas 78245, USA; 5 Department of Central Laboratory, Shaanxi Provincial People’s Hospital, Xi’an 710068, China

## Abstract

High blood pressure, or “hypertension,” is associated with high levels of oxidative stress in the paraventricular nucleus of the hypothalamus. While pomegranate extract is a known antioxidant that is thought to have antihypertensive effects, the mechanism whereby pomegranate extract lowers blood pressure and the tissue that mediates its antihypertensive effects are currently unknown. We have used a spontaneously hypertensive rat model to investigate the antihypertensive properties of pomegranate extract. We found that chronic treatment of hypertensive rats with pomegranate extract significantly reduced blood pressure and cardiac hypertrophy. Furthermore, pomegranate extract reduced oxidative stress, increased the antioxidant defense system, and decreased inflammation in the paraventricular nucleus of hypertensive rats. We determined that pomegranate extract reduced mitochondrial superoxide anion levels and increased mitochondrial function in the paraventricular nucleus of hypertensive rats by promoting mitochondrial biogenesis and improving mitochondrial dynamics and clearance. We went on to identify the AMPK-nuclear factor-erythroid 2 p45-related factor 2 (Nrf2) pathway as a mechanism whereby pomegranate extract reduces oxidative stress in the paraventricular nucleus to relieve hypertension. Our findings demonstrate that pomegranate extract alleviates hypertension by reducing oxidative stress and improving mitochondrial function in the paraventricular nucleus, and reveal multiple novel targets for therapeutic treatment of hypertension.

Many studies have focused on dietary and lifestyle interventions to manage hypertension^1^,^2^, a chronic disease in which arterial blood pressure is persistently elevated^3^. Consuming food with high antioxidant activity is known to mitigate the symptoms of hypertension^4^, and has been the subject of clinical trials^5^. The paraventricular nucleus region of the hypothalamus is an endocrine-autonomic control area of the brain that regulates sympathetic output and salt appetite^6^, and has been implicated in hypertension. Specifically, neuronal hyperactivity and oxidative stress within the paraventricular nucleus are thought to promote hypertension^7^.

The spontaneously hypertensive rat is a well-established model of hypertension and cardiovascular disease. Spontaneously hypertensive adult rats have systolic blood pressures ranging from 180 to 200 mmHg, and have significantly higher levels of oxidative stress and inflammation within the paraventricular nucleus^8^,^9^. Reducing oxidative stress and inflammation in the hypothalamus by antioxidant treatment has been shown to effectively lower blood pressure in an angiotensin II-induced rat model of hypertension^10^, suggesting that antioxidant-based reduction of oxidative stress and inflammation in the paraventricular nucleus may be an effective strategy for lowering blood pressure.

The peel, seeds and juice of the pomegranate fruit are abundantly rich in antioxidants. Punicalagin, a molecule known to have anti-inflammatory, anti-tumor and anti-diabetic effects^11^,^12^,^13^, is a major bioactive constituent of pomegranates^14^,^15^. The health benefits of punicalagin are thought to derive from its antioxidant role as a scavenger and ferrous chelator of hydrogen peroxide^13^. We have previously reported that pomegranate extract-enriched punicalagin promotes mitochondrial function by decreasing oxidative stress in a rat model of obesity-associated nonalcoholic fatty liver disease^16^. In addition, pomegranate extract reduces systolic and diastolic blood pressure and increases antioxidant activity in hemodialysis patients^17^, and protects the liver against acetaminophen-induced toxicity^11^.

Despite the fact that pomegranate extract/punicaligin has promising roles as an antioxidant, anti-inflammatory, and antihypertensive, the mechanism whereby pomegranate extract exerts it effects is unknown^4^,^5^. Understanding how pomegranate extract effects the central nervous system is critical for the rational design of hypertension therapy. Our data suggest that pomegranate extract reduces hypertension by lowering oxidative stress and improving mitochondrial function in the paraventricular nucleus, and that pomegranate extract-induced reduction of oxidative stress is mediated by the AMPK-Nrf2 pathway.

## Results

### Pomegranate extract reduces blood pressure and heart hypertrophy in spontaneously hypertensive rats

**Figure 1:**
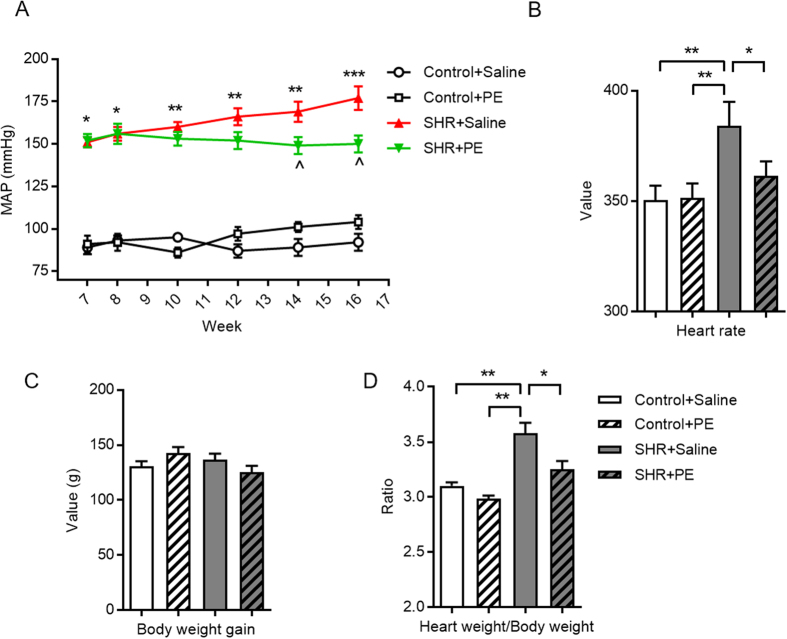
Effects of pomegranate extract supplementation on mean arterial pressure, body weight gain, and heart weight in hypertensive and control rats. Spontaneously hypertensive and control rats were administered either saline or pomegranate extract (150 mg/kg/day) for eight weeks. (**A**) The mean arterial pressure in control and hypertensive rats. *p < 0.05, **p < 0.01 and ***p < 0.001, ^*p* < 0.05. Asterisks indicate significantly elevated blood pressure of hypertensive rats versus controls. Arrows indicate significantly reduced blood pressure in hypertensive rats treated with pomegranate extract versus hypertensive rats treated with saline. (**B**) Heart rate in control and hypertensive rats. (**C**) Body weight gain in control and hypertensive rats. (**D**) Heart weight/body weight ratio. Values are the mean ± SEM from 10 animals. *p < 0.05, **p < 0.01. SHR, spontaneously hypertensive rats; PE, pomegranate extract.

Throughout our studies, spontaneously hypertensive rats and controls (Wistar-Kyoto, referred to hereafter as “control”) were treated with pomegranate extract or saline, respectively, for eight weeks. To determine if pomegranate extract reduces hypertension in this model, we first recorded systolic and diastolic blood pressures using a rat tail artery blood pressure test system, and calculated mean arterial pressure. Mean arterial pressure of hypertensive rats treated with saline increased over time compared to controls, but remained at a stable, lower level in the pomegranate extract treated group (Fig. 1A). Heart rates of pomegranate extract-treated hypertensive rats were also significantly lower at the end of treatment than the saline-treated group (Fig. 1B). While total body weight gain before or after pomegranate extract treatment did not differ between the groups (Fig. 1C), pomegranate extract reduced the higher heart weight to body weight ratio, an indicator of cardiac hypertrophy, in hypertensive rats, indicating that pomegranate extract treatment decreases hypertension-induced cardiac hypertrophy (Fig. 1D).

### Pomegranate extract inhibits superoxide anion and neuronal hyperactivity in the paraventricular nucleus of hypertensive rats

**Figure 2:**
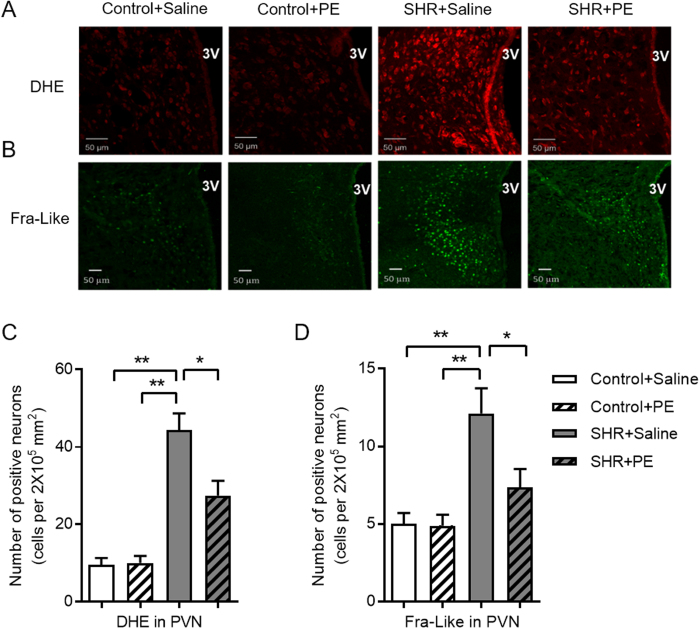
Effects of pomegranate extract supplementation on superoxide anion and chronic neuronal activation in the hypothalamic paraventricular nucleus of control and spontaneously hypertensive rats. Spontaneously hypertensive rats and controls were administered either saline or pomegranate extract (150 mg/kg/day) for eight weeks, sacrificed, and frozen sections were prepared from the paraventricular nucleus. (**A**) A representative immunofluorescence image showing superoxide production as detected with dihydroethidium staining (DHE, red fluorescence). (**B**) A representative immunofluorescence image showing Fra-Like expression (green fluorescence). Statistical analysis of dihydroethidium (**C**) and Fra-Like (**D**) positive neurons in the paraventricular nucleus of hypertensive and control rats, with or without pomegranate extract treatment. Values are the mean ± SEM from 10 animals. *p < 0.05, **p < 0.01. 3V, third ventricle; SHR, spontaneously hypertensive rats; PE, pomegranate extract, PVN, paraventricular nucleus.

We next determined if pomegranate extract reduces hypertension-induced oxidative stress in the paraventricular nucleus. Based on dihydroethidium staining, a dye widely used for measuring superoxide, we observed that pomegranate extract significantly reduced superoxide generation in the paraventricular nucleus of hypertensive rats (Fig. 2A,C), but had no effect on oxidative stress in controls.

Having established that pomegranate extract reduces levels of hypertension-induced superoxide in rats, we next investigated sympathetic nerve hyperactivity, a known consequence of oxidative stress in the hypothalamic paraventricular nucleus, and stimulator of hypertension^18^. We assessed neuronal activation based on Fra-like immunoreactivity, an indicator of chronic neuronal activation. We observed significantly higher levels of neuronal activity in hypertensive rats versus controls, which was significantly reduced by pomegranate extract treatment (Fig. 2B,D). Taken together, our data suggest that pomegranate extract decreases oxidative stress and chronic neuronal activation in the hypothalamic paraventricular nucleus of hypertensive rats.

### Pomegranate extract inhibits angiotensin converting enzyme expression in the paraventricular nucleus of hypertensive rats

**Figure 3:**
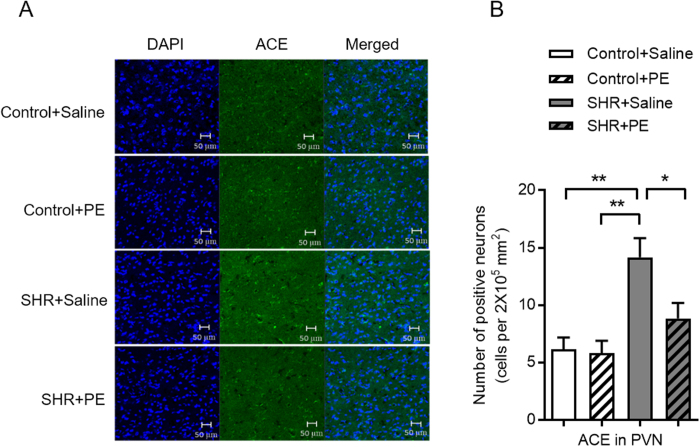
Effects of pomegranate extract supplementation on angiotensin converting enzyme expression in the hypothalamic paraventricular nucleus of control and hypertensive rats. Control and hypertensive rats were administered saline or pomegranate extract (150 mg/kg/day) for eight weeks, sacrificed, and frozen sections were prepared from the paraventricular nucleus. (**A**) Immunofluorescence of angiotensin converting enzyme-positive neurons (green fluorescence) in coronal sections of the paraventricular nucleus. (**B**) Statistical analysis of neurons that were positive for angiotensin converting enzyme expression. Values are the mean ± SEM from 8 animals. *p < 0.05, **p < 0.01. SHR, spontaneously hypertensive rats; PE, pomegranate extract; ACE, Angiotensin Converting Enzyme; PVN, paraventricular nucleus.

We next investigated the effects of pomegranate extract on the renin-angiotensin system, a known regulator of blood pressure. Components of the renin-angiotensin system, including angiotensin converting enzyme, are increased in the hypothalamic paraventricular nucleus of many animal models of hypertension^19^,^20^. We found significantly higher levels of angiotensin converting enzyme in the paraventricular nucleus of hypertensive rats compared to controls, and lower levels of angiotensin converting enzyme in hypertensive rats treated with pomegranate extract (Fig. 3A,B). These results suggest that pomegranate extract is effective in modulating the renin-angiotensin system in the paraventricular nucleus to relieve hypertension.

### Pomegranate extract elevates the antioxidant defense system in the paraventricular nucleus of hypertensive rats

**Figure 4:**
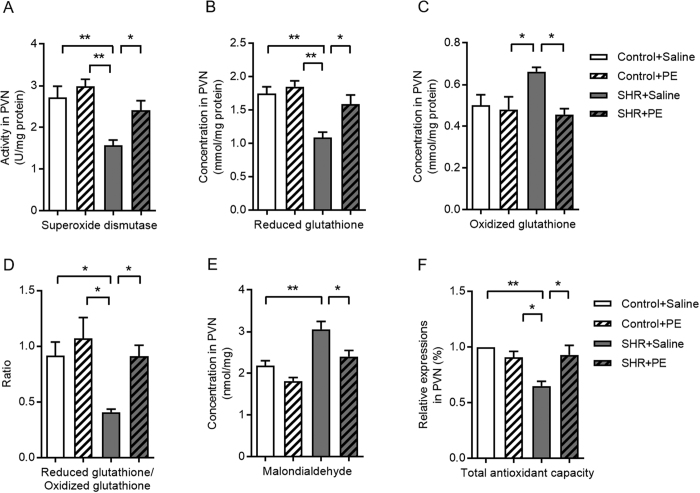
Effects of pomegranate extract supplementation on antioxidant capacity in the hypothalamic paraventricular nucleus of control and hypertensive rats. Control and hypertensive rats were administered either saline or pomegranate extract (150 mg/kg/day) for eight weeks, sacrificed, and homogenates were prepared from the paraventricular nucleus. (**A**) Superoxide dismutase activity, (**B**) reduced glutathione level, (**C**) oxidized glutathione level, (**D**) the ratio of reduced glutathione/oxidized glutathione, (**E**) lipid peroxidation malondialdehyde concentration, and (**F**) total antioxidant capacities were calculated using commercial kits. Values are the mean ± SEM from 8 animals. *p < 0.05, **p < 0.01. SHR, spontaneously hypertensive rats; PE, pomegranate extract; PVN, paraventricular nucleus.

Since we found that pomegranate extract reduces total levels of reactive oxygen species in hypertensive rats, and lack of antioxidant protection is known to increase formation of reactive oxygen species^21^,^22^, we next determined if pomegranate extract affects the antioxidant defense system in the context of hypertension. We measured both enzymatic and nonenzymatic antioxidant levels in the paraventricular nucleus of pomegranate extract-treated hypertensive rats and controls based on individual antioxidant proteins, malondialdehyde, an indicator of lipid oxidation, and total antioxidant capacity, an assay that detects a panel of antioxidant enzymes. Hypertensive rats had significantly more oxidative stress in the paraventricular nucleus based on lower levels of superoxide dismutase (Fig. 4A) and reduced glutathione (Fig. 4B), higher levels of oxidized glutathione (Fig. 4C), a reduced ratio of reduced glutathione to oxidized glutathione (Fig. 4D), increased malondialdehyde (Fig. 4E) and reduced total antioxidant capacity (Fig. 4F) compared to controls, clearly demonstrating that hypertension significantly increases oxidative stress in the paraventricular nucleus. All of these indicators of oxidative stress were significantly reversed by pomegranate extract treatment of hypertensive rats (Fig. 4A–F), suggesting that pomegranate extract elevates the antioxidant defense system to protect the paraventricular nucleus from oxidative stress.

### Pomegranate extract reduces inflammation in hypertensive rats

**Figure 5:**
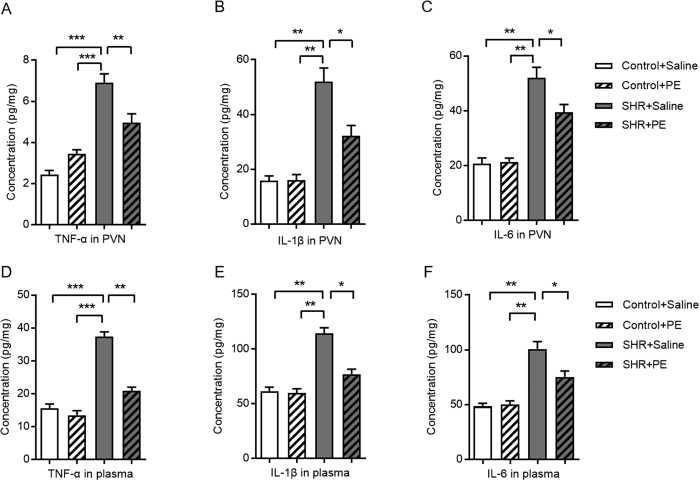
Effects of pomegranate extract supplementation on paraventricular nucleus and plasma inflammatory cytokines of control and hypertensive rats. Control and hypertensive rats were administered either saline or pomegranate extract (150 mg/kg/day) for eight weeks, sacrificed, tissue homogenates were prepared from the paraventricular nucleus, and plasma was collected. Pro-inflammatory cytokines (**A**) TNF-α, (**B**) IL-1β, and (**C**) IL-6 were measured in the paraventricular nucleus with commercial kits. (**D**) TNF-α, (**E**) IL-1β, and (**F**) IL-6 were measured in the plasma with commercial kits. Values are the mean ± SEM from 8 animals. *p < 0.05, **p < 0.01, ***p < 0.001. SHR, spontaneously hypertensive rats; PE, pomegranate extract; PVN, paraventricular nucleus.

Since oxidative stress is a known mediator of inflammation^23^, we next investigated inflammation in the context of pomegranate extract-mediated reduction of oxidative stress in hypertension. We detected significantly increased levels of pro-inflammatory cytokines, including TNF-α (Fig. 5A), IL-1β (Fig. 5B) and IL-6 (Fig. 5C), in the paraventricular nucleus of hypertensive rats versus controls based on ELISA. After pomegranate extract treatment, however, IL-1β, TNF-α and IL-6 were significantly decreased in the paraventricular nucleus of hypertensive rats (Fig. 5A–C). We observed similar results in plasma from pomegranate extract-treated hypertensive rats and controls (Fig. 5D–F). These data indicate that pomegranate extract effectively reduces hypertension-induced inflammation.

### Pomegranate extract improves mitochondrial function in the paraventricular nucleus of hypertensive rats

**Figure 6:**
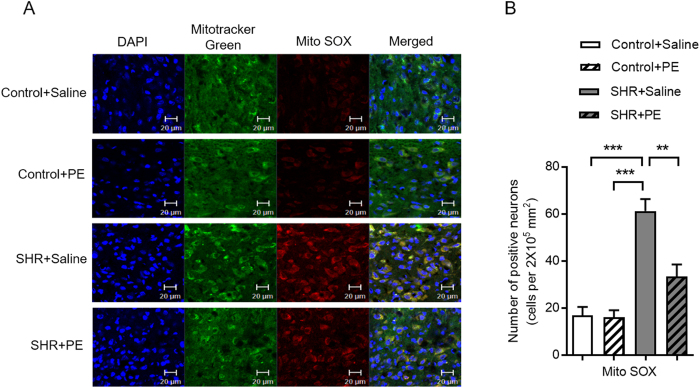
Effects of pomegranate extract supplementation on mitochondrial superoxide in the hypothalamic paraventricular nucleus of control and hypertensive rats. Control and hypertensive rats were administered either saline or pomegranate extract (150 mg/kg/day) for eight weeks, sacrificed, and frozen sections were prepared from the paraventricular nucleus. (**A**) Mitochondrial superoxide (red fluorescence) was measured using the fluorescent probe Mito SOX, which was detected with confocal microscopy. Mitotracker green was used to localize mitochondria. (**B**) Statistical analysis of the number of mitochondrial superoxide-positive neurons in control and hypertensive rats. Values are the mean ± SEM from 8 animals. **p < 0.01, ***p < 0.001. SHR, spontaneously hypertensive rats; PE, pomegranate extract.

Since oxidative stress is known to impair mitochondrial function^24^,^25^, we investigated mitochondrial function in the paraventricular nucleus of hypertensive rats. We first assessed superoxide levels in mitochondria of the hypothalamic paraventricular nucleus in rat brain slices using MitoTracker Green, a fluorescent dye that localizes to mitochondria, combined with MitoSOX Red, a fluorescent dye that detects mitochondrial superoxide. We found significantly higher levels of mitochondrial superoxide anion in the hypertensive rats compared to controls, which was reduced by pomegranate extract (Fig. 6A,B). Reduction of mitochondrial superoxide anion in hypertensive rats suggested to us that mitochondrial function may improve upon pomegranate extract treatment.

**Figure 7:**
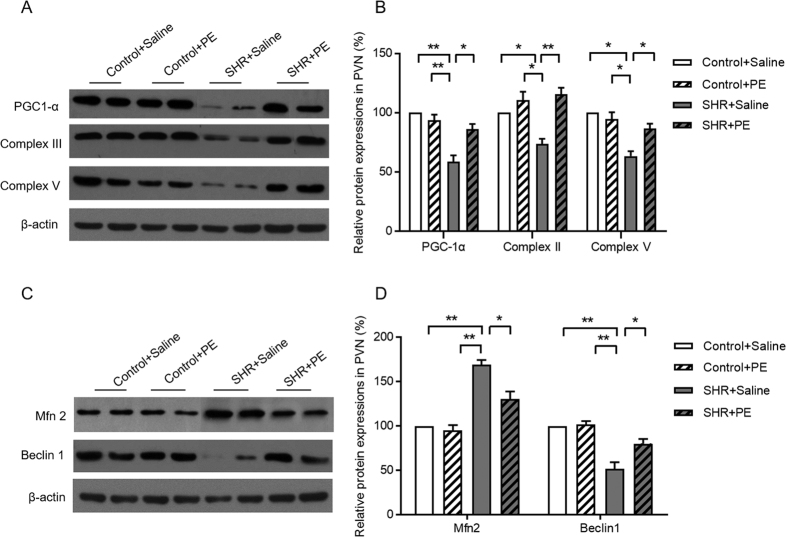
Effects of pomegranate extract supplementation on the expression of mitochondrial related proteins in the hypothalamic paraventricular nucleus of control and hypertensive rats. Control and hypertensive rats were administered either saline or pomegranate extract (150 mg/kg/day) for eight weeks, sacrificed, and lysates were prepared from the paraventricular nucleus for Western blotting. (**A**,**B**) Expression levels are shown for the mitochondrial biogenesis proteins PGC-1α, Complex III and V subunits (**A**, Western blotting image; **B**, statistical analysis). (**C**,**D**) Expression levels are shown for the mitochondrial dynamics proteins, Mfn2 and Beclin1 (**C**, Western blotting image; **D**, statistical analysis). Values are the mean ± SEM from 8 animals. *p < 0.05, **p < 0.01. SHR, spontaneously hypertensive rats; PE, pomegranate extract; PVN, paraventricular nucleus; PGC-1α, proliferator-activated receptor gamma co-activator.

We next investigated mitochondrial biogenesis, a strong indicator of mitochondrial function^26^, in the context of hypertension. Compared to controls, hypertensive rats had significantly lower levels of proteins that are critical for mitochondrial biogenesis, including proliferator-activated receptor gamma co-activator (PGC-1α) and mitochondrial respiratory chain Complex III and Complex V (Fig. 7A,B). Pomegranate extract treatment increased levels of PGC-1α, Complex III and Complex V in hypertensive rats, suggesting that pomegranate extract alleviates hypertension-induced reduction of mitochondrial biogenesis (Fig. 7A,B).

A balance between mitochondrial fission and fusion, or “mitochondrial dynamics”, is required for proper mitochondrial function. To determine if hypertension alters mitochondrial dynamics, we measured levels of Mfn2, a mitochondrial membrane protein that promotes mitochondrial fusion and contributes to maintenance and operation of the mitochondrial network^27^, in hypertensive rats. We found that Mfn2 levels were increased in the paraventricular nucleus of hypertensive rats compared to controls, indicating that hypertension promotes mitochondrial fusion. Treatment of hypertensive rats with pomegranate extract significantly decreased Mfn2 levels (Fig. 7C,D), suggesting that pomegranate extract improves mitochondrial dynamics.

**Figure 8:**
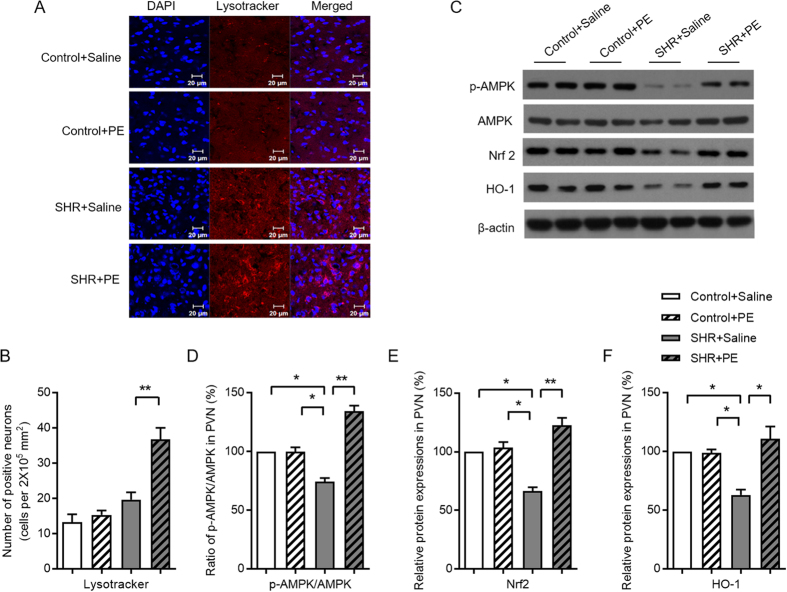
Effects of pomegranate extract supplementation on mitophagy and the expression of AMPK-Nrf2 pathway components in the hypothalamic paraventricular nucleus of control and hypertensive rats. (**A**) Mitophagy (red fluorescence) was measured using the fluorescent probe Lysotracker, which was detected with confocal microscopy. (**B**) Statistical analysis of the number of the mitophagy-positive neurons. (**C**,**D**,**E**,**F**) The protein expression levels of AMPK, p-AMPK, Nrf2 and hemeoxygenase were analyzed by Western blotting (**B**, Western blotting image; **C**,**D**,**E**, statistical analysis). Values are the mean ± SEM from 8 animals. **p < 0.01, ***p < 0.001. SHR, spontaneously hypertensive rats; PE, pomegranate extract; PVN, paraventricular nucleus; Nrf2, nuclear factor-erythroid 2 p45-related factor 2; HO-1, hemeoxygenase.

As a final indicator of mitochondrial function, we determined how pomegranate extract effects mitophagy, the process of removing impaired or abnormal mitochondria from the cell, in hypertensive rats. We found that levels of Beclin1, a key regulator of mitophagy, were reduced in the paraventricular nucleus of hypertensive rats, but were restored upon pomegranate extract treatment, suggesting that pomegranate extract promotes the clearance of dysfunctional mitochondria in the context of hypertension (Fig. 7C,D). We also found a significant increase in Lysotracker Red, an additional indicator of mitophagy, in hypertensive rats treated with pomegranate extract (Fig. 8A,B). Taken together, our data suggest that hypertension impairs mitochondrial function and clearance in the paraventricular nucleus, and that pomegranate extract alleviates hypertension-induced mitochondrial dysfunction and clearance.

### Pomegranate extract acts via the AMPK-Nrf2 pathway to reduce oxidative stress in the paraventricular nucleus of hypertensive rats

We next determined the mechanism whereby pomegranate extract reduces oxidative stress and improves mitochondrial function in the paraventricular nucleus of hypertensive rats. AMPK is a phosphorylation-dependent metabolic master switch that controls mitochondrial biogenesis^28^. Phosphorylated AMPK activates Nrf2, which promotes expression of antioxidant proteins, including hemeoxygenase, that protect against oxidative damage triggered by injury and inflammation^29^. We found significantly lower levels of p-AMPK in the paraventricular nucleus of hypertensive rats, which was reversed after pomegranate extract treatment (Fig. 8C,D). Likewise, Nrf2 and hemeoxygenase protein levels increased in response to pomegranate extract treatment of hypertensive rats (Fig. 8C,E,F). These data suggest that pomegranate extract-induced reduction in oxidative stress and improvement of mitochondrial function in the paraventricular nucleus of hypertensive rats is mediated by AMPK phosphorylation and downstream Nrf2/hemeoxygenase signaling.

## Discussion

In this study, we have investigated the mechanism of action of pomegranate extract-mediated alleviation of hypertension. Using a spontaneously hypertensive rat model, we have found that pomegranate extract activates AMPK in the paraventricular nucleus of the hypothalamus, which stimulates Nrf2/hemeoxyganse signaling, causing scavenging of free radicals and reduction of oxidative stress, which improves mitochondrial function. In addition to lowering hypertension-induced oxidative stress, we found that pomegranate extract decreases chronic sympathetic activity in the paraventricular nucleus. Hypertension-related sympathetic overdrive in the hypothalamic paraventricular nucleus is thought to result from high levels of oxidative stress^6^, and clearance of free superoxide radicals in the paraventricular nucleus lowers excessive sympathetic activity and arterial blood pressure in hypertensive rats^30^. We thus propose that pomegranate extract-induced reduction of sympathetic overdrive in hypertensive rats is due to the effects of pomegranate extract as an antioxidant.

Oxidative stress in the hypothalamic paraventricular nucleus is also associated with the production of pro-inflammatory cytokines^31^, and reducing hypertension in rats is known to decrease pro-inflammatory cytokine levels in plasma^32^. Furthermore, activation of NF-κB, a pro-inflammatory signaling protein, in the paraventricular nucleus increases oxidative stress and excess sympathetic excitation, while inhibition of NF-κB reduces both pro-inflammatory cytokine levels and oxidative stress, and alleviates hypertension^9^. In our study, pomegranate extract treatment decreased pro-inflammatory cytokines in hypertensive rats, suggesting that an imbalance between pro- and anti-inflammatory cytokines in the paraventricular nucleus could contribute to hypertension-induced sympathetic excitation.

Impaired mitochondrial function increases oxidative stress, which causes organ damage in hypertension. Antioxidants provide promising strategies to decrease hypertension-mediated mitochondrial dysfunction and organ damage. In our current study, chronic pomegranate extract administration significantly reduced production of mitochondrial superoxide anion in hypertensive rats. Furthermore, pomegranate extract significantly improved mitochondrial function in hypertensive rats based on assays measuring mitochondrial biogenesis (PGC-1α, Complex III and Complex V), mitochondrial dynamics (fusion-related protein Mfn2), and mitochondrial clearance (mitophagy-related protein Beclin1).

We then investigated the AMPK-Nrf2 pathway, a known sensor of metabolic stress, as a mechanism whereby pomegranate extract decreases oxidative stress in the context of hypertension. Oxidative stress causes phosphorylation of AMPK^29^, which activates Nrf2, which increases hemeoxygenase, a potent antioxidant enzyme^29^. The spontaneously hypertensive rats are associated with impaired Nrf2 signaling, inflammation, and oxidative stress, which can be restored by administration of resveratrol^33^. Impaired Nrf-2-regulated mitochondrial biogenesis in rostral ventrolateral medulla contributes to hypertension under systemic inflammation^34^. Hemeoxygenase is a known target of punicalagin, and punicalagin-induced hemeoxygenase expression is dramatically blocked after specific inhibition of Nrf2^35^. We found that hypertensive rats treated with pomegranate extract had increased levels of phosphorylated AMPK, Nrf2 and hemeoxygenase protein compared to controls, suggesting that pomegranate extract acts through the AMPK-Nrf2 pathway to reduce hypertension-induced oxidative stress and downstream mitochondrial dysfunction.

Punicaligin is the main antioxidant compound in pomegranate peels and has been the topic of many studies^14^. Punicalagin has a neuroprotective effect in ischemia-induced brain injury, where it decreases both malondialdehyde and mitochondria-generated reactive oxygen species, and increases enzymes that protect against oxidative stress^12^. We have previously shown that punicalagin promotes Nrf2 protein expression and nuclear translocation followed by induction of antioxidative enzymes including hemeoxygenas^36^. Of the polyphenols found in pomegranates, punicalagin is the most abundant ellagitannin, a class of proteins with known antioxidant properties. We have previously shown that pomegranate extract gavage causes a time-dependent increase in serum punicalagin levels, suggesting that punicalagin circulates in the body to exert its beneficial effects. We have previously shown that pomegranate extract and exercise have an additive effect in improving immune function in high-fat-diet fed rats by inhibiting inflammatory cytokine secretion and decreasing oxidative stress^37^. As in the previous study of our group^16^,^36^,^38^, we used pomegranate extract containing 40% punicalagin and showed that punicalagin has been identified as the major active component. While we did not test pure punicaligin directly in the present study, our data are consistent with punicalagin as the primary active component in pomegranate extract that acts to decrease oxidative stress in the paraventricular nucleus of hypertensive rats. Future experiments will be carried out with the pure punicalagin in our system. Additionally, another possible mechanism, the regulating effect on mitophagy by pomegranaate metabolite Urolithin A, should be further studies^39^.

In conclusion, these results obtained, together with previous studies of our group^16^,^36^,^38^,^40^,^41^,^42^, may suggest that pomegranate extract alleviates symptoms of hypertension by reducing oxidative stress, improving mitochondrial function, and decreasing chronic activation of the sympathetic nervous system in the paraventricular nucleus, and have identified the AMPK-Nrf2 pathway as a mechanistic link between pomegranate extract-induced reduction of oxidative stress. Our studies thus reveal novel insights into the mechanism of action of pomegranate extract as an antihypertensive therapy, and identify additional new targets for the therapeutic treatment of hypertension.

## Materials and Methods

### Experimental design

Ten-week-old male spontaneously hypertensive rats and Wistar-Kyoto rats (used as the control of spontaneously hypertensive rats) were purchased from Charles River Laboratory Animal, Ltd. (Beijing, China). Wistar-Kyoto control rats were administered pomegranate extracts or normal saline; spontaneously hypertensive rats were administered pomegranate extracts or normal saline. The rats were housed in individual standard cages on a 12 hour light/12 hour dark cycle in a temperature-controlled (21 ± 2.0 °C) environment for a week of acclimatization, with ad libitum access to water and a commercial diet. After adapting to the environment for one week, the rats were administered pomegranate extracts (purchased from Tianjing Jianfeng Natural Product R&D Co. Ltd., China, containing 40% punicalagin, at a dose of 150 mg/kg/day, diluted into 2 ml of saline) or an equal volume of saline by gavage for eight weeks. The growing area of pomegranate is in Shaanxi province of China. The main composition are polyphenol and punicalagin. The extract preparation method was mainly column chromatography. The animal experiments were performed in accordance with the Guide for the Care and Use of Laboratory Animals (National Institutes of Health publication No. 85-23, revised 1996) and were approved by the Institutional Animal Care and Use Committees of Xi’an Jiaotong University.

At the end of the eight weeks, all rats were weighed and intraperitoneally injected with ketamine (80 mg/kg) and acepromazine (10 mg/kg) as anesthesia. Blood was sampled from the carotid artery. The brain was removed and fixed in formaldehyde solution for one week, deposited in 30% sucrose solution for 2–3 days, and then thin-sectioned for use in further experiments. Another portion of rats were sacrificed, and the paraventricular nucleus of the hypothalamus was rapidly dissected from fresh brain and stored in a tube at −80 °C.

### Blood pressure measurement

Blood pressure and heart rate were measured each week at 8:00–10:00 am in conscious rats using a noninvasive tail-cuff system (NIBP, AD Instruments, Australia). The temperature of the incubator was adjusted to 36 °C with floor heating. The blocker and sensor were successively set on the tail of awake rats in the incubator. Rats were allowed to calm, at which time the cuff pressure was increased for blood pressure measurement. The blocker deflates and decompresses until there is a systolic blood pressure pulse, which was recorded as the systolic blood pressure. The pressure continues to decrease, and there is a peak, which was recorded as the diastolic blood pressure. The mean arterial pressure was calculated as diastolic blood pressure+(systolic blood pressure-diastolic blood pressure)/3, and the final value was the mean value of five measurements.

### Microscopic anatomy of the hypothalamic paraventricular nucleus

Palkovits’ micro-dissection procedure was employed to isolate the paraventricular nucleus from rat brains. Briefly, the brain was serially sectioned into 300 mm slices, from the bregma to lambda, with a cryostat. The sections were transferred to coverslips and placed on a cooling stage maintained at −80 °C. The paraventricular nucleus of the hypothalamus was punched with the help of a stereotaxic atlas. The micro-dissected paraventricular nuclei were then stored at −80 °C until future analysis.

### Fluorescence staining

#### Preparation of the paraventricular nucleus slice

The frozen paraventricular nucleus slice was equilibrated for 10 min at room temperature and permeated for 30 min with 0.3% Triton x-100 at 37 °C. The slice was then washed with PBS three times for 5 min.

#### Detection of angiotensin-converting enzyme or superoxide anion

The prepared paraventricular nucleus slice was incubated with dihydroethidium (diluted to 1:200, Santa Cruz) for 20 min at 37 °C. After washing with PBS three times, 5 min each time, the slice was mounted and observed with a confocal laser scanning microscope (Zeiss 510, Germany).

#### Detection of the expression of Fra-Like or angiotensin-converting enzyme

The prepared paraventricular nucleus slice was incubated with primary antibody (anti-Fra-Like or anti-angiotensin converting enzyme, diluted to 1:200, Santa Cruz) overnight at 4 °C. The slice was washed three times with PBS, 5 min each time, and was then incubated with FITC-conjugated secondary antibody (diluted to 1:200, Santa Cruz) for 1 h at 37 °C. After washing, the slice was observed with a confocal laser scanning microscope.

#### Detection of mitochondrial superoxide anion

The prepared paraventricular nucleus slice was incubated with 50 nM MitoTracker Green and then 1 μM Mito SOX (Invitrogen, Molecular Probes, Carlsbad, CA) for 20 min. The slice was washed three times with PBS, 5 min each time. DAPI was applied to stain cell nuclei (diluted to 1:300, Invitrogen). After washing, the slice was observed with a confocal laser scanning microscope.

### Antioxidant capacity detection

The paraventricular nucleus was homogenized, and the indicators for oxidative stress and antioxidant capacity in paraventricular nucleus homogenate were analyzed, including superoxide dismutase, reduced glutathione, oxidized glutathione, the ratio of reduced glutathione/oxidized glutathione, lipid peroxidation malonaldehyde, and total antioxidant capacity with commercial kits (Jiancheng Bioengineering Institute, Nanjing, China) in accordance with the manufacturer’s instructions.

### Western blotting

The paraventricular nucleus was lysed with IP lysis buffer (Beyotime, Nanjing, China) and then homogenized. The protein in the supernatant was quantified with a BCA assay kit (Pierce, Rockford, IL, USA), which was performed according to the manufacturer’s instructions. Equal amounts of protein were denatured and then analyzed with polyacrylamide gel electrophoresis. The separated proteins were transferred to a PVDF membrane. The membrane was blocked with plasma. Primary antibodies included anti-PGC-1α, anti-Mfn2, anti-Nrf2, anti-hemeoxygenase, anti-β-actin (Santa Cruz), anti-OxPhos Complex III or anti-Complex V (Invitrogen), anti-Beclin1, anti- AMPK, and anti-p-AMPK (Thr 172, Cell Signaling Technology, Beverly, MA). Primary antibodies were added and incubated with the membrane. After washing, the membrane was incubated with secondary antibody, and signals were observed by chemiluminescence. The result was photographed and analyzed with NIH Image J analysis software.

### ELISA for cytokine assessment

The levels of IL-1β, TNF-α and IL-6 in the extracted tissue and plasma were measured with a commercially available enzyme-linked immunosorbent assay kit (R&D Systems, Minneapolis, MN) according to the manufacturer’s instructions. The optical density of each well was determined using a microplate reader (Bio-Rad, Hercules, CA).

### Statistical analysis

The values are presented as the mean ± SEM. The data were analyzed using a Student’s t-test for two-group comparisons or a one-way ANOVA followed by the Newman–Keuls correction for multiple comparisons. Blood pressure data were analyzed by repeated measures ANOVA. GraphPad Prism 5.0 statistical and graphing software was used for the statistical analyses. P values < 0.05 were considered statistically significant.

## Additional Information

**How to cite this article**: Sun, W. *et al*. Pomegranate extract decreases oxidative stress and alleviates mitochondrial impairment by activating AMPK-Nrf2 in hypothalamic paraventricular nucleus of spontaneously hypertensive rats. *Sci. Rep*. **6**, 34246; doi: 10.1038/srep34246 (2016).
